# Potential Nexus of Non-alcoholic Fatty Liver Disease and Type 2 Diabetes Mellitus: Insulin Resistance Between Hepatic and Peripheral Tissues

**DOI:** 10.3389/fphar.2018.01566

**Published:** 2019-01-14

**Authors:** Wan Mu, Xue-fang Cheng, Ying Liu, Qian-zhou Lv, Gao-lin Liu, Ji-gang Zhang, Xiao-yu Li

**Affiliations:** ^1^Department of Clinical Pharmacy, Shanghai General Hospital, Shanghai Jiao Tong University School of Medicine, Shanghai, China; ^2^Department of Pharmacy, Zhongshan Hospital, Fudan University, Shanghai, China

**Keywords:** non-alcoholic fatty liver diseases, diacylglycerols, PKC𝜀, hepatic insulin resistance, type 2 diabetes mellitus

## Abstract

The liver is the central metabolic organ and plays a pivotal role in regulating homeostasis of glucose and lipid metabolism. Aberrant liver metabolism promotes insulin resistance, which is reported to be a common characteristic of metabolic diseases such as non-alcoholic fatty liver disease (NAFLD) and type 2 diabetes mellitus (T2DM). There is a complex and bidirectional relationship between NAFLD and T2DM. NAFLD patients with hepatic insulin resistance generally share a high risk of impaired fasting glucose associated with early diabetes; most patients with T2DM experience non-alcoholic fatty liver (NAFL), non-alcoholic steatohepatitis (NASH), and other more severe liver complications such as cirrhosis and hepatocellular carcinoma (HCC). Additionally, hepatic insulin resistance, which is caused by diacylglycerol-mediated activation of protein kinase C epsilon (PKC𝜀), may be the critical pathological link between NAFLD and T2DM. Therefore, this review aims to illuminate current insights regarding the complex and strong association between NAFLD and T2DM and summarize novel and emerging targets for the treatment of hepatic insulin resistance based on established mechanistic knowledge.

## Introduction

Recent researches have shown an increasing global incidence of obesity-related metabolic diseases with the high prevalence of sedentary behavior and high fat and calorie diets (Ng et al., 2014). Nutritional excess is a major forerunner of metabolic disease, enhancing the secretion of insulin from pancreatic β cells while attenuating the metabolic actions of insulin in the liver, skeletal muscle, and adipose tissue (Wilcox, 2005). As a common pathological feature among metabolic diseases, including obesity, non-alcoholic fatty liver disease (NAFLD), and type 2 diabetes mellitus (T2DM) (Tilg et al., 2017), insulin resistance decreases the metabolic response of target cells to insulin, resulting in an impaired ability of circulating or injected insulin to decrease blood glucose levels at the whole-organism level (Reaven, 2005). Insulin resistance conventionally refers to impaired glucose uptake dependent on glucose transporter of insulin 4 (GLUT4) in skeletal muscle and adipose tissue (Zisman et al., 2000). Mice studies have proved that single knockout of insulin receptor in skeletal muscle or adipose tissue is insufficient to produce glucose tolerance abnormalities or insulin resistance syndrome (Bluher et al., 2002; Long et al., 2011). However, liver-specific insulin receptor knockout (LIRKO) mice exhibit severe insulin resistance and dramatic glucose intolerance (Michael et al., 2000). Thus, hepatic insulin resistance, which is characterized by increased hepatic glucose production, might be a fundamental cause of fasting hyperglycemia contributing to the development of T2DM (Lallukka and Yki-Jarvinen, 2016).

NAFLD has begun to be recognized as a clinical entity in the 1980s (Ludwig et al., 1980), and has been defined as a spectrum of progressive liver diseases encompassing simple fatty infiltration in >5% of hepatocytes (steatosis), fatty infiltration plus inflammation (NASH), fibrosis, and cirrhosis in the absence of excessive alcohol consumption (Anstee et al., 2011). NAFLD patients usually have hepatic insulin resistance, which is associated with NAFLD-related lipid accumulation (especially diacylglycerols, DAGs), inflammation, endoplasmic reticulum (ER) stress, and oxidative stress. Moreover, hepatic insulin resistance is the key cause of impaired fasting glucose, which contributes substantially to the development of T2DM (Pansuria et al., 2012). It is reported that NASH is the progressive form of NAFLD that can lead to liver fibrosis and cirrhosis, with subsequent complications such as hepatocellular carcinoma (HCC). Compared with non-NAFLD-associated HCC, a significant number (41.7%) of NAFLD-associated HCC have no underlying cirrhosis and exhibited a higher prevalence of metabolic features (T2DM, dyslipidemia, coronary artery disease) in a clinical trial (Ertle et al., 2011). Thus HCC, as the main cause of liver disease-related deaths (Yatsuji et al., 2009), may not always occur in the setting of cirrhosis, and patients with metabolic diseases such as NAFLD, T2DM are more sensitive to HCC.

Taken together, reducing hepatic lipid accumulation and improving hepatic insulin resistance might be effective ways to prevent the progression of NAFLD to T2DM. However, the exact mechanisms underlying these pathological processes are not entirely understood. A comprehensive understanding of insulin resistance and the temporal and mechanistic connections between NAFLD and T2DM will provide a scientific basis to further explore new therapeutic targets for the treatment of metabolic diseases. Here, we discussed the crosstalk of the energy metabolism and pathological molecular pathways between hepatic and extrahepatic insulin resistance, and summarized some new pharmacological strategies targets for the treatment of NAFLD and hepatic insulin resistance (Table 1), which is intended to protect patients with NAFLD from the onset of T2DM and other potential pathological consequences (e.g., liver fibrosis and cirrhosis and HCC).

**Table 1:** Potential and emerging targets for the treatment of non-alcoholic fatty liver disease (NAFLD) and hepatic insulin resistance.

Pharmacological targets	Therapeutic drug or drug class	Research object pre-clinical models/clinical trials	Main results	Reference
GLP-1R	GLP-1R agonists	Mice with NAFLD Healthy humans or patients with NASH	Promote increased energy expenditure; activate brown adipose tissue; reduce hepatic glucose production and hepatic lipid content; decrease white adipose and liver lipid synthesis	Campbell and Drucker, 2013; Seghieri et al., 2013; Armstrong et al., 2016
PPAR γ	PPAR γ agonist Thiazolidinediones (TZDs)	Mice with NAFLD Patients with NASH and Prediabetes or T2DM	Activate PPARγ to improve insulin-mediated suppression of adipocyte lipolysis; lower rates of post-prandial fatty acid turnover; decrease ectopic lipid accumulation to improve hepatic and muscle insulin resistance	Phielix et al., 2011; Cusi et al., 2016
PPAR α/δ	PPAR α/δ dual agonists Elafibranor (GFT505)	Mice with NAFLD Patients with NASH	Promote fatty acid oxidation; decrease hepatic *de novo* lipogenesis and inflammation; improve hepatic and peripheral insulin sensitivity in diet-induced NAFLD	Staels et al., 2013; Pawlak et al., 2015; Ratziu et al., 2016
FXR	FXR agonists BAR502	Mice with NAFLD FXR-knockout (KO) mice Patients with NASH	FXR agonists repress bile acid synthesis and hepatic gluconeogenesis; decrease hepatic DAG by activating diacylglycerol kinases to ameliorate lipid-induced hepatic insulin resistance. BAR502, a dual FXR and GPBAR1 agonist, protects against hepatic steatosis, hepatic inflammation, and glucose intolerance caused by High Fat Diet	Cai and Sewer, 2013; Kliewer and Mangelsdorf, 2015; Carino et al., 2017
MGAT	MGAT1/2/3 inhibitors	Obese (DIO) mice Patients with NAFLD	MGAT1 inhibitors suppress the conversion of monoacylglycerols to DAGs; normalize glucose tolerance; decrease PKC𝜀 activation; improve hepatic insulin signaling. MGAT2 inhibitors prevent diet-induced obesity and hepatic steatosis	Hall et al., 2012, 2014; Okuma et al., 2015; Yang and Nickels, 2015
DGAT	DGAT 1/2 inhibitors	DGAT1 knockout mice Obese (DIO) mice Healthy humans	Suppress acylation of DAGs into triglycerides; primarily reduce intestinal lipid absorption by increasing intestinal fatty acid oxidation and GLP-1 secretion; prevent weight gain, hepatic steatosis, and insulin resistance	Cao et al., 2011; Maciejewski et al., 2013; Tomimoto et al., 2015
Liver mitochondrial uncoupling	Liver-targeted mitochondrial uncoupling agents (DNP-ME and CRMP)	Mice with lipodystrophy-associated NASH and diabetes Rats with NAFLD and T2DM	Increase hepatic mitochondrial energy expenditure; reduce hypertriglyceridemia and hepatic steatosis; reduce hepatic DAGs-PKC𝜀 activity and hepatic acetyl-CoA content to reverse hepatic insulin resistance	Perry et al., 2013, 2015b; Abulizi et al., 2017
Pask	Pask inhibitors	Mice with NAFLD	Decrease liver triglyceride accumulation; reduce insulin resistance; ameliorate obesity	Wu et al., 2014; Zhang et al., 2015a,b


## Liver as the Core Organ Regulating Glucose and Lipid Metabolism

### Regulation of Glucose and Lipid Metabolism in the Liver

The liver is a crucial metabolic organ for regulating glucose and lipid homeostasis, as well as meet energy needs in response to different metabolic stresses. Metabolic activities of the liver are strictly controlled by a variety of metabolic substrates such as free fatty acids (FFAs), hormones, and neuronal signals (Rui, 2014). Especially, insulin, which is the only hormone that decreases blood glucose, is critical for both carbohydrate and lipid metabolism *in vivo*. During the transition from a fasted state to a fed state, increased blood glucose stimulates the secretion of insulin, promoting glycogen synthesis and lipogenesis but suppressing gluconeogenesis in the liver, thereby maintaining the normal range of blood glucose levels *in vivo* (Kubota et al., 2017). However, the effect of insulin is repressed in insulin-resistant individuals, and persistent hepatic glucose output contributes to post-prandial hyperglycemia, which is a critical characteristic of metabolic diseases.

**FIGURE 1:**
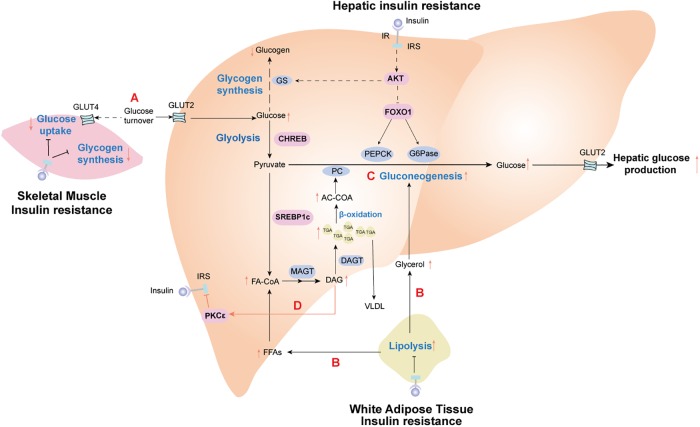
Insulin resistance effect profiles in the metabolic cross-talk network between liver and peripheral tissues. **(A)** Skeletal muscle insulin resistance impairs insulin-stimulated muscle glucose uptake, resulting in increased glucose delivery to the liver. **(B)** Adipose insulin resistance impairs insulin-mediated suppression of lipolysis, leading to the release of glycerol and fatty acid (FAs). These nutrients are further redirected to the liver, driving hepatic lipid synthesis and activating hepatic gluconeogenesis. **(C)** In the liver, increased fatty acid oxidation activates hepatic gluconeogenesis via acetyl-CoA-mediated activation of pyruvate carboxylase (PC), while glycerol delivery to the liver increases gluconeogenesis via a substrate push. **(D)** Diacylglycerol (DAG)-mediated activation of protein kinase C epsilon (PKC𝜀) impairs hepatic insulin signaling, thereby constraining insulin-stimulated hepatic glycogen synthesis. Hepatic lipid synthesis continues unabated. The net results of these changes are the root cause of hepatic selective insulin resistance, which is characterized by decreased hepatic glycogen synthesis and increased hepatic gluconeogenesis and hepatic lipid synthesis. IR, insulin receptor; IRS, insulin receptor substrate; PEPCK, phosphoenolpyruvate carboxykinase; G6Pase, glucose 6-phoshase. Solid black lines stand for the normal metabolic pathways. Black dotted lines and red solid lines represent the metabolic pathways of pathological states.

Insulin resistance is specific to the liver and peripheral organs. For example, peripheral insulin resistance primarily occurs in muscle and adipose tissue and is characterized by disturbed insulin-mediated stimulation of glucose uptake and utilization but increased fat decomposition of adipose tissue. However, selective hepatic insulin resistance is characterized by insufficient suppression of hepatic gluconeogenesis, decreased glycogen synthesis, and increased lipid accumulation (Wilcox, 2005). Over many decades, numerous studies have shown that peripheral insulin resistance leads to reduced glucose uptake from the circulation attributable to an increased conversion of incoming glucose to the liver. For a more comprehensive understanding of the pathological processes associated with insulin resistance, we discuss the metabolic links and interactions between hepatic and extrahepatic insulin resistance below (Figure 1).

### Insulin Resistant Metabolic Cross-Talk Network Between Liver and Peripheral Tissues

Target tissues can have differential responses to insulin, such that skeletal muscle is more sensitive to insulin than the liver and adipose tissue (Mizuno et al., 2004; Petersen et al., 2007). Therefore, insulin resistance in skeletal muscle is a defect observed during the early development of T2DM and often occurs before insulin resistance in the liver and adipose tissue (Martin et al., 1992; Zierath and Wallberg-Henriksson, 2002); further, it may involve abnormal metabolism in other tissues. Upon high carbohydrate consumption, humans with insulin resistance exhibit reduced uptake of glucose and muscle glycogen synthesis, as well as a doubling of both liver triglyceride levels and hepatic *de novo* lipogenesis without any changes in circulating adipokines (Petersen et al., 2007). These data indicate that insulin resistance in the skeletal muscle shifts post-prandial energy storage from muscle glycogen to hepatic lipid storage. As such, insulin resistance in the skeletal muscle contributes to increasing post-prandial blood glucose, which increases glucose uptake in the liver. Elevated liver glucose concentrations activate the carbohydrate response element-binding protein (ChREBP), which stimulates intracellular glycolysis, thereby providing metabolic precursor materials for *de novo* lipogenesis. Thus, insulin resistance in skeletal muscle indirectly contributes to increased *de novo* lipogenesis in NAFLD (Roden, 2006; Figure 1).

Adipose tissue serves as the body’s energy stores and actively takes in superfluous blood glucose to store excess energy in the form of triglycerides during conditions of nutritional excess. Insulin resistance weakens insulin-mediated suppression of lipolysis in adipose tissue, resulting in the release of a large amount of FFAs and glycerol into the circulation. Increased circulating FFAs provide the primary source of non-esterified fatty acids for liver triglycerides synthesized by hepatocyte in NAFLD patients (Rui, 2014). What’s worse, FFAs can activate the intrinsic apoptosis pathway in hepatocytes via c-Jun N-terminal kinase (JNK), which promotes progression from simple steatosis to NASH and even advanced hepatic fibrosis (Wree et al., 2011). Moreover, adipocyte-specific adipokines secreted by visceral adipose tissue, such as adiponectin and leptin, seem to has a close correlation with the development of NAFLD (Wree et al., 2014). Lipodystrophy, characterized by a loss of adipose tissue, often occurs in patients with adipose IR, which may be due to a decreased capacity to synthesize and store triglycerides for adipose cells. This is the principal contributor to excess storage of ectopic fat accumulation in the liver of NAFLD patients (Tilg et al., 2017; Figure 1). Leptin has been reported to reduce both hepatic and intramyocellular lipid content in mice and humans with lipodystrophy, which mostly could be attributed to reduction in caloric intake, with concomitant improvement in both hepatic and peripheral insulin sensitivity (Shimomura et al., 1999; Petersen et al., 2002).

Overall, it is well established that the adipocyte secretome, including FFAs and adipokines, is closely linked to the pathogenesis development of NAFLD. Understanding the metabolic cross-talk of adipose tissue and liver as part of a broader metabolic disorder is likely to improve the management of patients with liver disease.

## Hepatic Lipid Metabolism and Insulin Resistance

### Pathophysiological Aspects of Hepatic Lipids Metabolism and Insulin Resistance in NAFLD

#### Intrahepatic Triglyceride Accumulation

Intrahepatic triglyceride accumulation, or steatosis, is a hallmark of NAFLD that develops when the rate of fatty acid input (fatty acid uptake from plasma and *de novo* lipogenesis) exceeds the rate of fatty acid output (fatty acid oxidation and secretion of triglycerides in the form of very-low-density lipoproteins). Intrahepatic triglyceride content is a better predictor of hepatic insulin resistance than visceral adiposity or body mass index in individuals with NAFLD. Furthermore, interventions that reverse intrahepatic triglyceride accumulation are associated with amelioration of hepatic insulin resistance in NAFLD patients and rodent models of this disease (Petersen et al., 2005; Perry et al., 2013, 2015b).

Experiments measuring hepatic DAGs content and hepatic insulin sensitivity in humans have indicated that DAGs strongly correlate with hepatic insulin resistance (Kumashiro et al., 2011; Ter Horst et al., 2017), whereas other potential mediators of hepatic insulin resistance (such as ceramides) show an inconsistent relationship (Luukkonen et al., 2016; Ter Horst et al., 2017). Increased hepatic DAGs activate protein kinase C𝜀 (PKC𝜀) (Jornayvaz and Shulman, 2012), which impairs the tyrosine kinase activity of the insulin receptor by inhibiting phosphorylation of the insulin receptor at Thr1160 (Petersen et al., 2016; Figure 1). These substantial and available data provide a putative causal link between increased intrahepatic triglyceride content and hepatic insulin resistance.

#### Inflammation

The pathogenesis development of NAFLD was described by the theory “two hits” hypothesis proposed by Day and James (1998). The first “hit” is deposition of free fatty acid and triglyceride in hepatocytes (steatosis), and the second “hit” is the progression of steatosis to NASH (Day and James, 1998). Histopathologically, NASH was defined as fatty infiltration plus inflammation (Anstee et al., 2011). It has been demonstrated that pro-inflammatory cytokines can interfere with insulin signaling by activating various inflammatory pathways in preclinical models of NASH (Tilg and Diehl, 2000). Classic inflammatory pathways that induce insulin resistance encompass the inhibitor kappa B kinase beta/nuclear factor kappa B (IKKβ/NF-κB) pathway and the c-Jun N-terminal kinase/activator protein 1 (JNK/AP1) pathway (Johnson and Olefsky, 2013). There are many pro-inflammatory cytokines, including interleukin-6 (IL-6) and tumor necrosis factor α (TNF-α), that can activate intracellular kinases such as JNK and IKKβ. Activation of downstream transcription factors (i.e., AP-1 and NF-κB) results in the inhibition of insulin receptor signaling by means of serine phosphorylation of insulin receptor substrate 1/2 (Gao et al., 2002).

Adipose tissue has emerged as a major source of circulating inflammatory cytokines. Concentrations of specific cytokines, such as IL-1β or IL-6, are expressed 10-fold to 100-fold higher in adipose tissue than in the human liver (Moschen et al., 2010, 2011). Therefore, high concentrations of circulating inflammatory signals might induce hepatic insulin resistance via inflammatory pathways, thereby providing a positive feedback loop that amplifies liver inflammation. Furthermore, there are other factors that can activate intrahepatic inflammatory pathways resulting in insulin resistance, including microbiota-derived lipopolysaccharide (LPS), FFAs, and advanced glycation end products (Catrysse and van Loo, 2017). FFAs might substantiate insulin resistance, leading to lysosomal instability with leakage of cathepsin B and induction of the IKKβ/NF-κB pathway; alternatively, the caspase-1-IL-1β/IL-18 pathways might be activated through the NACHT, LRR, and PYD domains-containing protein 3 (NALP3) inflammasome (Tilg et al., 2017). In addition, Kupffer cells, as the resident macrophages of liver, would be activated to release cytokines and chemokines, recruit new macrophages or other immune cells, in response to exogenous and endogenous pro-inflammatory molecular signals (e.g., LPS, excess FFAs, cytokines). Therefore, these cells critically contribute to hepatic inflammation in the progression of NASH. Also, Kupffer cells are known to switch from an anti-inflammatory ‘M2’ state to a proinflammatory ‘M1’ state, probably inducing insulin resistance through interactions with hepatocytes (Odegaard et al., 2008). This effect was probably mediated through TNFα secretion, as inhibition of TNFα attenuated the effect of Kupffer cells on hepatocytes (Huang et al., 2010).

Currently, it remains unclear at which sites inflammatory processes are initiated. Therefore, clinical evidence for a major role of inflammation in insulin resistance is still in its infancy.

#### Endoplasmic Reticulum (ER) Stress and Oxidative Stress

Hepatic insulin resistance is also associated with other pathological processes of NAFLD that might contribute to dysregulated glucose metabolism. ER stress, which refers to activation of the unfolded protein response attributable to the accumulation of newly synthesized unfolded proteins, has recently been proposed to play a crucial role in hepatic steatosis (Zhang et al., 2014). During over-nutrition conditions of hyperlipidemia and hyperglycemia, hepatocytes are confronted with high rates of protein synthesis, resulting in activation of the ER stress response. It has been shown that ER stress drastically contributes to hepatic insulin resistance by inducing inflammatory responses involving NF-κB and JNK signaling, which further affect insulin signaling (Ozcan et al., 2004; Grootjans et al., 2016).

In addition, a recent study reported a compensatory upregulation of hepatic mitochondrial respiration in obese individuals with fatty liver; however, this adaptation is abolished in obese individuals with NASH (Koliaki et al., 2015). To explain this exciting discovery, Koliaki et al. showed that during hepatic lipid accumulation, hepatic mitochondria transiently adapt to increased lipid availability by upregulating their oxidative capacity. However, excessive lipids impair antioxidant capacity and accelerate oxidative stress with mitochondrial leakage or increased reactive oxygen species levels, resulting in aggravated inflammation and insulin resistance in NASH patients (Satapati et al., 2015).

Overall, improvements in our understanding of the effects of hepatic lipid metabolism on hepatic insulin resistance might inform potential therapeutic strategies for protecting NAFLD patients from T2DM. Further discussions regarding the physiological and pathophysiological regulation of hepatic glucose metabolism in insulin resistant livers are necessary.

### “Selective” Hepatic Insulin Resistance

Under the hyperglycemic fed condition, activated hepatic insulin signaling inhibits hepatic glucose production and promotes hepatic lipogenesis. Individuals with T2DM manifest selective hepatic insulin resistance in which insulin fails to suppress gluconeogenesis but continues to activate lipogenesis, producing the deadly combination of hyperglycemia and hypertriglyceridemia. Thus, T2DM patients usually exhibit the classic triad of hyperinsulinemia, hyperglycemia, and hypertriglyceridemia. However, initial research by Biddinger showed hyperglycemia and hyperinsulinemia but not hypertriglyceridemia in LIRKO mice (Biddinger et al., 2008).

To further explain the essential mechanisms of hepatic insulin resistance in T2DM patients and LIRKO mice, a “pathway-selective hepatic insulin resistance” hypothesis has been proposed (Leavens and Birnbaum, 2011); this hypothesis explains the hepatic metabolic state of synchronous-enhanced hepatic glucose production and lipid synthesis. Hepatic insulin signaling can directly suppress gluconeogenesis by activating RAC-α serine/threonine-protein kinase (also known as AKT or PKB), which can result in phosphorylation and exclusion of Forkhead box (Foxo1) from the nucleus of the hepatocyte and consequent transcription-mediated reduction in hepatic gluconeogenesis (Haeusler et al., 2014). Otherwise, expression of sterol regulatory element binding-protein 1c (SREBP-1c) in the *de novo* lipogenesis pathway can be enhanced through Akt-dependent activation of mammalian target of rapamycin complex 1 (mTORC1) and inhibition of Foxo1, which are both sufficient for *de novo* lipogenesis (Caron et al., 2015; Titchenell et al., 2016). The “pathway-selective hepatic insulin resistance” hypothesis states that during hepatic insulin resistance, Akt does not sufficiently activate Foxo1 to suppress gluconeogenesis; however, Akt maintains activation of the mTORC1 protein-kinase complex and the sterol regulatory element-binding protein 1c (SREBP-1c) transcription factor to enhance lipid synthesis. Interestingly, it has been reported that double knockout mice lacking Akt and Foxo1 suppress hepatic glucose production normally during a hyperinsulinemic-euglycemic clamp (Lu et al., 2012). Furthermore, insulin suppresses hepatic glucose production within minutes of administration *in vivo*, which is unlikely to be explained via transcriptionally mediated processes.

Whether insulin resistance is selectively imposed during gluconeogenesis while leaving its actions on lipogenesis intact is still under investigation; however, careful examination of the available data indicate that, to a great extent, hepatic insulin resistance is driven by complex crosstalk and modulation of metabolic fluxes between the liver and extrahepatic tissues (Otero et al., 2014; Samuel and Shulman, 2016; Titchenell et al., 2016; Figure 1).

Titchenell et al. (2015) has proved that the abnormal hepatic glucose output and insulin resistance resulting from liver-specific ablation of insulin receptor was largely rescued by Foxo1 deletion, despite lack of autonomous liver insulin signaling. Consequently, there must exist an extrahepatic mechanism to regulate hepatic glucose production independent of hepatic Akt/Foxo1 insulin signaling pathway. Furthermore, Perry et al. (2015a) used a comprehensive metabolomics flux approach to demonstrate that suppression of hepatic glucose production by insulin is temporally associated with decreased white adipose tissue lipolysis-derived hepatic acetyl CoA. Hepatic acetyl CoA, which is produced via β-oxidation of fatty acids in the mitochondria of hepatocytes, is known as an allosteric activator of pyruvate carboxylase (PC). When PC activity is increased, the transition from pyruvate to glucose is catalyzed during hepatic gluconeogenesis. To some extent, it provides evidence for a liver autonomous mechanism to receive the signal initiated by insulin’s interaction with a non-hepatic tissue. Perry et al. (2015a) also suggested that macrophage-induced white adipose tissue lipolysis leading to increased hepatic acetyl CoA content and increased PC activity is a key molecular mechanism linking inflammation of white adipose tissue to both fasting and post-prandial hyperglycemia in T2DM (Figure 1).

## Hepatic Insulin Resistance: a Key Nexus of NAFLD and T2DM

With the global trend of obesity, the incidence of NAFLD in obesity has rapidly risen to almost 70% and is recognized as the hepatic component of metabolic syndrome (Streba et al., 2015). A recent prospective study showed that NAFLD occurs in more than 70% of patients with T2DM (Loomba et al., 2012) and could be regarded as a risk factor for T2DM independent of age and other factors such as obesity (Lallukka and Yki-Jarvinen, 2016). NAFLD often precedes T2DM, and patients with NAFLD almost always exhibit hepatic insulin resistance, which might be the critical factor driving the development of the pathogenesis from NAFLD to T2DM.

### Hepatic Insulin Resistance in NAFLD

Hepatic insulin resistance is a complex phenomenon in patients with NAFLD. Although multiple pathological mechanisms have been proposed for hepatic insulin resistance in NAFLD (as reviewed above), hepatic insulin resistance is almost universally associated with intrahepatic accumulation of triglyceride and DAGs, with the latter activation of PKC𝜀 and subsequent inhibition of insulin-stimulated insulin receptor kinase activity in a variety of experimental and clinical models (Jornayvaz and Shulman, 2012; Birkenfeld and Shulman, 2014; Perry et al., 2014; Figure 1). The diacylglycerol-PKC𝜀 hypothesis of hepatic insulin resistance has recently been validated in humans with NAFLD (Kumashiro et al., 2011). Additionally, DAGs-induced hepatic insulin resistance is attributable to compartmentation of DAGs in the cytosolic and membrane compartments (Kumashiro et al., 2011). Knock-down mice for CGI-58, a lipase activator, show increased DAGs accumulation in lipid droplets, whereas DAGs accumulation is prevented in the cell membrane. This, in turn, prevents PKC𝜀 translocation to the cell membrane and protects against intrahepatic DAGs-induced hepatic insulin resistance (Brown et al., 2010). Nevertheless, CGI-58 antisense oligonucleotide data clearly indicate that dissociation of NAFLD and hepatic insulin resistance is likely attributable to insufficient activation of PKC𝜀 by DAGs that are sequestered in lipid droplets. Future work will better discern the importance of specific lipid compartments in the pathogenesis of insulin resistance.

### Hepatic Insulin Resistance: A Key Pathophysiology Driving T2DM

Type 2 diabetes mellitus is a progressive metabolic disease characterized by insulin resistance and a significant decline in beta cell function (Lillioja et al., 1993). In the early stage of insulin resistance, blood-glucose increases and stimulates insulin secretion by islet beta cells. Thus, insulin resistant individuals experience compensatory hyperinsulinemia. Once the islet β cells cannot secrete enough insulin to compensate for the defect in insulin action, T2DM might occur in insulin resistant individuals (Kahn et al., 2014).

Hepatic insulin resistance is somewhat peculiar as the effects of hepatic insulin signaling result in insufficient suppression of hepatic gluconeogenesis and decreased glycogen synthesis but increased lipid accumulation. This selective hepatic insulin resistance contributes to simultaneous increases in liver glucose production and fat synthesis, resulting in hyperglycemia and dyslipidemia characteristic of T2DM. Studies using genetic models of tissue-specific insulin resistance obtained by selectively knocking out insulin receptor genes with Cre-loxP technology have found differing effects on insulin resistance in different tissues during systemic metabolic disease (Kubota et al., 2017). The results show that muscle insulin receptor knockout (MIRKO) mice or fat insulin receptor knockout (FIRKO) mice still have normal blood glucose and insulin levels, as well as normal glucose tolerance test responses, although they have separately exhibited specific metabolic abnormalities of tissue-specific insulin resistance (Biddinger and Kahn, 2006). In contrast, LIRKO mice show severe insulin resistance, fasting and post-prandial hyperglycemia, glucose intolerance, and hyperinsulinemia. These studies indicate that insulin resistance of peripheral tissues alone is not enough to cause abnormality of glucose tolerance or insulin resistance syndrome. However, hepatic insulin resistance as the leading cause of fasting hyperglycemia might be the critical factor driving the development of T2DM.

## New Perspectives in the Treatment of T2DM: Targeting Regulation of Hepatic Metabolism

Collectively, NAFLD-related pathophysiology includes hepatic ectopic fat deposition, inflammation, ER stress, and oxidative stress (Haas et al., 2016), all of which aggravate hepatic insulin resistance and promote hyperglycemia, hyperlipidemia, and other metabolic disorders. Metabolic disorders that include systemic glucose and lipid metabolism show a progressive exacerbation, resulting in the occurrence of T2DM. Currently, therapeutic strategies for treating NAFLD primarily encompass limiting caloric intake and proper exercise to maintain a healthy lifestyle. However, the standard treatment for NAFLD has not been approved in current clinical practice guidelines (Nascimbeni et al., 2013).

Some potential pharmacological target strategies are emerging to influence the energy balance, inhibit key enzymes involved in lipid synthesis or metabolic pathways that contribute to NAFLD, such as agonists for Peroxisome proliferator-activated receptors (PPARs, e.g., PPARγ, PPAR α/δ) and Farnesoid X receptor (FXR), and analogs for Glucagon-like peptide-1 receptors (GLP-1R); inhibitors of monoacylglycerol acyltransferase (MGAT) and diacylglycerol acyltransferase (DGAT) (Table 1). Besides, a novel class of liver-targeted mitochondrial uncoupling agents increases hepatocellular energy expenditure, reversing the metabolic and hepatic complications of NAFLD [e.g., 2,4-dinitrophenol-methyl ether (DNP-ME) controlled-release mitochondrial protonophore (CRMP)] (Table 1). Finally, our laboratory has been working on studies of mechanisms of lipid metabolic disorders and related targets of therapeutic drugs. Our previous research shows that nuciferine and siRNA PAS-domain containing protein kinase (Pask, an evolutionarily conserved nutrient-responsive protein kinase) could alleviate the accumulation of lipogenesis, inflammation, and oxidative stress in NAFLD (Zhang et al., 2015b). Considering the complex and bidirectional relationship between NAFLD and T2DM, we speculate that Pask plays a potential role in the deterioration from NAFLD to T2DM (Zhang et al., 2015a), which will be addressed in our future research of nuciferine-mediated anti-diabetic effect (Table 1).

Taken together, the relationship between NAFLD and T2DM is complex and bidirectional. NAFLD provides the necessary biological milieu for development of T2DM (Lallukka and Yki-Jarvinen, 2016), and the presence of T2DM increases the risk of liver diseases (Raff et al., 2015), with the potential to progress to NASH, cirrhosis and, in some patients, HCC (Koehler et al., 2016; Kwok et al., 2016). However, it is remarkable that existing guidelines do not advocate screening for liver-related complications in patients with T2DM, making the liver a potentially neglected organ during the progression of chronic metabolic diseases. Therefore, solidifying a robust overall paradigm regarding the pathological mechanisms of liver metabolism in NAFLD and T2DM would contribute to a search for potential therapies targeting hepatic steatosis and lipid-induced hepatic insulin resistance. It is also of great clinical importance to advocate for a more active and systematic surveillance of NAFLD in patients with T2DM, with a view toward potential early treatment (Vizuete et al., 2017).

## Author Contributions

WM and X-yL designed the research. X-fC and YL consulted and helped to categorize related references. WM and X-yL wrote the manuscript. G-lL and Q-zL participated in the revision of the manuscript. X-yL and J-gZ supervised the research. All authors contributed to and approved the final version of the manuscript.

## Conflict of Interest Statement

The authors declare that the research was conducted in the absence of any commercial or financial relationships that could be construed as a potential conflict of interest.
